# Antibiotic resistance and molecular characterization of clinical *Shigella* spp. isolated from hospitals located in Tehran and Qazvin cities, Iran

**DOI:** 10.1186/s13104-025-07113-6

**Published:** 2025-03-11

**Authors:** Termeh Raoufian, Babak Pakbin, Akram Sadat Tabatabaee Bafroee

**Affiliations:** 1 Department of Biology, East Tehran Branch, Islamic Azad University, Tehran, Iran; 2 Werner Siemens Chair of Synthetic Biotechnology, Dept. of Chemistry, Technical University of Munich, 85748 Munich, Bavaria Germany

**Keywords:** *Shigella*, Pulse field gel electrophoresis, Genotyping, Multidrug resistance

## Abstract

**Objective:**

This research aims to investigate the epidemiology and genetic changes of *Shigella* species in the cities of Qazvin and Tehran from 2003 to 2023, with a focus on assessing antibiotic resistance among the isolates. This study is based on the analysis of 80 *Shigella* isolates, which were obtained from patients’ feces.

**Results:**

Using pulsed-field gel electrophoresis (PFGE) as the primary typing method, the isolates were classified into 13 distinct clusters, revealing a dominant pattern that has persisted for over twenty years. This persistence may be attributed to factors such as poor hygiene, personal contact, immigration, and antibiotic resistance. Genetic analysis indicated clonal expansion among the isolates over the two-decade period. Notably, the highest resistance was observed against amoxicillin, with several isolates exhibiting multidrug resistance.

## Introduction

The genus *Shigella* is the typical main cause of diarrheal diseases which is close to *Escherichia coli* [1] belonging to the *Enterobacteriaceae* family [2]. The *Shigella* genus is one of the most common diarrhoea-causing pathogens in Asia and is responsible for shigellosis, an intestinal infection that is also known as bacillary dysentery [3]. Shigellosis is a non-systemic, intestinal, and acute infection characterized by the destruction of the colonic epithelium responsible for bloody diarrhoea, which is sometimes accompanied by mucus, abdominal pain, and fever [4]. The main virulence factors of *Shigella* are carried by the *inv* plasmid (a 200-kb virulence plasmid that contains the essential genetic information for invasion). The *ipa*H gene involved in *Shigella* invasion is encoded by both bacterial plasmid and chromosome. This would make it possible to identify *Shigella* spp. through the use of polymerase chain reaction (PCR) by amplification of the *ipa*H genetic marker. *Shigella* has a low infectious dose and is one of the 4 causes of moderate to severe diarrhoea in children aged 12 to 59 months [5].

There are four fecal-oral ways to transmit this disease through hands, contaminated food or water, through transmission by flies, and sexual transmission [6]. The symptoms of shigellosis and the severity of shigellosis depend on the infecting species and the characteristics of the host [7]. The high prevalence of shigellosis in poor countries is generally attributed to the lack of clean water, poor sanitation, malnutrition, and the cost of antibiotic treatment. In addition, the effect of shigellosis is intensified due to the emergence of strains resistant to several drugs, which have jeopardized antibiotic treatment [8].

The spread of antimicrobial resistance has increased since the 1960s and is a major problem in global public health. Antibiotic resistance of intestinal pathogens, especially *Shigella* species, is a critical problem worldwide [9]. The development of antibiotic resistance in bacteria has led to an increased failure rate in the treatment of infectious diseases caused by bacteria, as drugs that were previously sensitive to them are no longer effective. Bacteria can be resistant due to their inherent properties or they can achieve this resistance capacity by mutation and gene transfer. Different mechanisms of antibiotic resistance include poor penetration of drugs into the cell, the release of antibiotics by efflux pumps, modification of the target by mutation, and hydrolysis of antibiotics [10].

The World Health Organization (WHO) recommends ciprofloxacin as first-line antimicrobial therapy for all shigellosis patients with dysentery, regardless of age. However, excessive use or misuse of antibiotics in the treatment of shigellosis increases antibiotic resistance and limits treatment options for *Shigella* infections [3].

Typing of bacterial strains, or identification of bacteria at the strain level, is especially important for the diagnosis, treatment, and epidemiological monitoring of bacterial infections. In recent years, various molecular or genetic techniques, including techniques related to PCR, RFLP, plasmid profiling, etc., have been used to classify bacteria and microbes [11, 12].

Among them, PFGE is considered the “gold standard” for bacterial typing. This technique involves the enzymatic restriction of bacterial DNA, followed by the isolation of the resulting DNA fragments using a pulsed-field electrophoresis chamber. Subsequently, bacteria are assigned to clones based on PFGE banding patterns. PFGE is one of the best genotyping methods that is used for typing and epidemiological studies in different regions. This method can be used for all human pathogens with its high reproducibility and differentiation power.

Therefore, in the present study, 102 *Shigella* isolates collected between 2002 and 2022 from Tehran and Qazvin were genotyped by PFGE method and their drug susceptibility against 13 different antibiotics was also determined.

## Materials and methods

### Bacterial isolation and identification

*Shigella* isolates (*n* = 80) previously isolated from stool samples of 1-32-year-old shigellosis patients (Table 1) were collected from microbiology laboratories of hospitals in Iran (Qazvin–Tehran) between 2002 and 2023. These isolates were presumably identified as *Shigella* spp. using Microbiological (colony morphology and Gram staining) and standard biochemical tests (such as citrate tests, urase, TSA, indole, MRVP, and motility tests) [13]. Then all *Shigella* isolated were molecularly confirmed by amplification of the *ipa*H gene using PCR technique and specific primers [14, 15].

**Table 1:** Demographic information of isolates

Demographic information of isolates					
Age range	Sample code	Separation time	Source	City	Organization
< 8	1–43	2018–2023	Stool	Tehran	The microbial collection of Pars Research laboratory
1–32	44–83	2003–2023	Stool	Tehran	Milad hospital
< 2	84–102	2018–2023	Stool	Qazvin	The specialized laboratory of Qazvin University of Medical Sciences and Medical Services

### Typing of isolates by PFGE technique

Pulse Net by PFGE standardized protocol was used for subtyping of *Shigella* isolates. Chromosomal DNA was prepared using the method described by Kaufman with some modifications [14]. In short, bacteria cultured on plates were resuspended in a buffer solution (1 mol/L Tris: 0.5 mol/L EDTA, pH 8.0) and adjusted to achieve absorbance values ranging from 0.8 to 1.0 at a wavelength of 610 nm. After which agarose plugs were prepared using Low melting point agarose 2% and proteinase K. Cells in the agarose plugs were lysed by treatment with a lysis solution (1 mol/L Tris, 0.5 mol/L EDTA (pH 8.0), 1% sarcosine, and 0.5 mg of proteinase K) for 24 h at 56 °C. Four washing steps were performed, with Tris-EDTA (TE) buffer (10 mmol/L Tris, 1 mmol/L EDTA, pH 8.0). Forty units of *Xba*I restriction enzyme (Roche Diagnostic GmbH, Mannheim, Germany) were applied for plugs in a freshly prepared buffer and incubated for 24 h. *Xba*I digested *Salmonella enterica* serotype Braenderup H9812 plugs were used as DNA molecular weight size markers. The electrophoresis was performed with CHEF Mapper XA System (Bio-Rad) and consisted of 6 V at 14 °C for 18 h with the increasing pulsed time from 2.16 s to 54.17 s, including angel of 120° and actual current of 110–120 A [14, 16].

### Analysis of PFGE patterns

PFGE patterns were analyzed by GelCompare II version 4.0 software (Applied Maths, Sint-Matenslatem, Belgium) and the patterns were compared by using the Dice coefficient and UPGMA (unweighted pair group method with arithmetic averages) clustering. A dendrogram was constructed using an optimization value of 1.0% and a position tolerance of 2.0%. Isolates with ≥ 90% similarity were considered as a single cluster. Assigning the types and interpretation of PFGE-generated patterns was performed according to the guidelines set by Eftekhari and colleagues [17].

### Antibiotic susceptibility test

The antibiotic susceptibility of these isolates was determined using a disc diffusion method, according to the guidelines of the Clinical Laboratories Standards Institute (CLSI) [18]. Thirteen antimicrobial agents (Padtan teb Co. Iran) were used including ampicillin (AM, 10 µg), amikacin (AN, 30 µg), kanamycin (K, 30 µg), chloramphenicol (C, 30 µg), gentamicin (GM, 10 µg), nalidixic acid (NA, 30 µg), tetracycline (TE, 30 µg), imipenem (IMP, 10 µg), azithromycin (AZM, 15 µg), amoxicillin (AMX, 30 µg), cefepime (FEP, 30 µg), cefoxitin (CTX, 30 µg) and norfloxacin (NOR, 10 µg). The *Escherichia coli* strain ATCC 25,922 was employed as a reference strain.

### Statistical analysis

The data analysis for this study was conducted using the statistical software package SPSS, specifically version 22. The chi-square test was used to calculate the associations between studied resistance genes and antibiotic resistance and the Cramer’s-V test was also used to measure the strength of the association. The *P*-value was set at less than 0.05.

## Result

### Bacterial isolation

In total, 80 isolates were identified and confirmed as *Shigella* spp. through microbiological, biochemical, and molecular tests.

### Genetic relatedness analysis

**Fig. 1:**
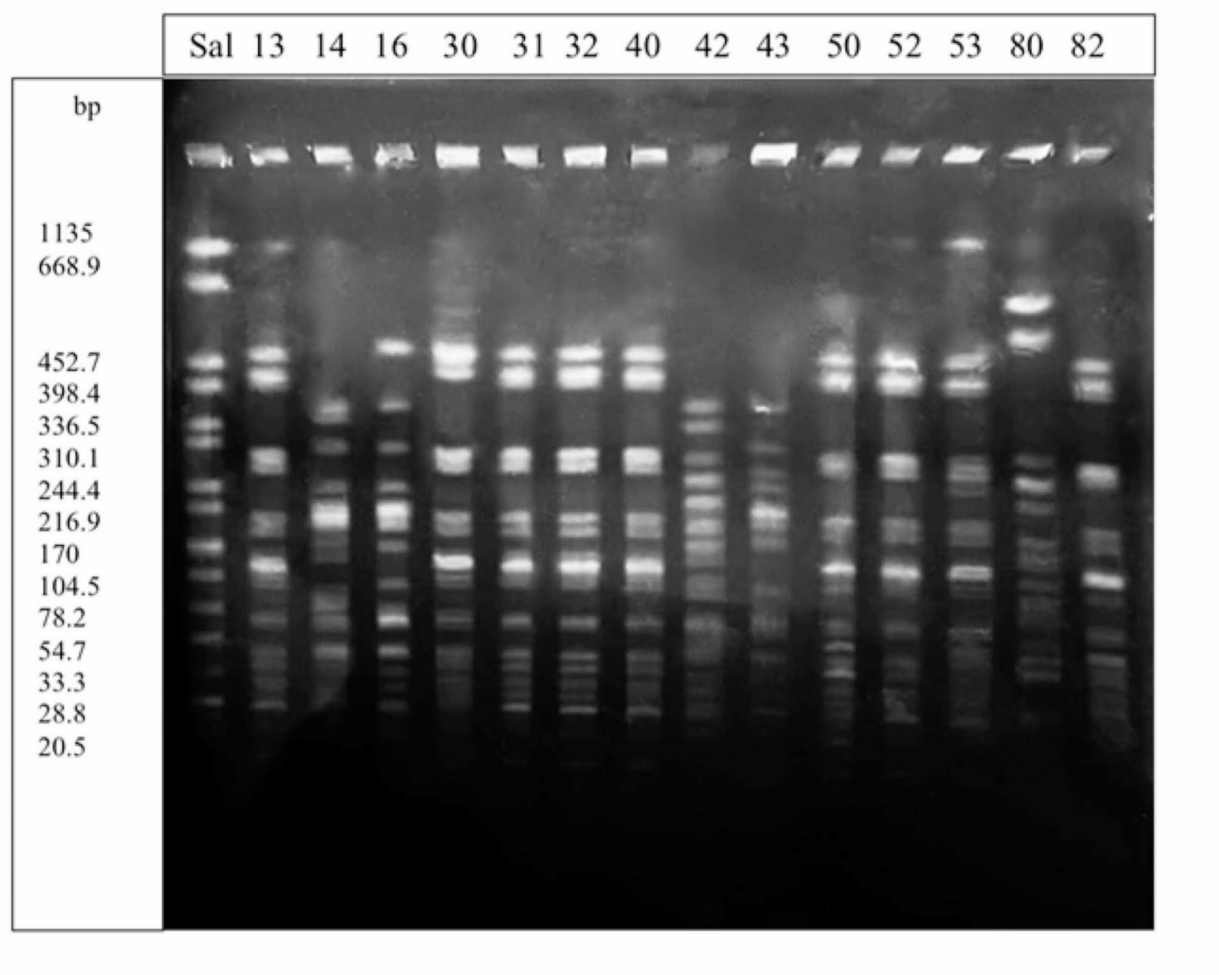
Xba1 macro-restriction patterns obtained for *Shigella* after the plug was lysed in a modified lysis buffer. H9812 strain of *Salmonella* serotype Braenderup was used as a molecular marker. First line: Marker, second to fifteenth line: *Shigella* isolates the size of the Salmonella marker bands is plotted from left to right: 452.7, 398.4, 336.5, 310.1, 244.4, 216.9, 170, 104.5, 78.2, 54.7, 33.3, 28.8, 20.5

**Fig. 2:**
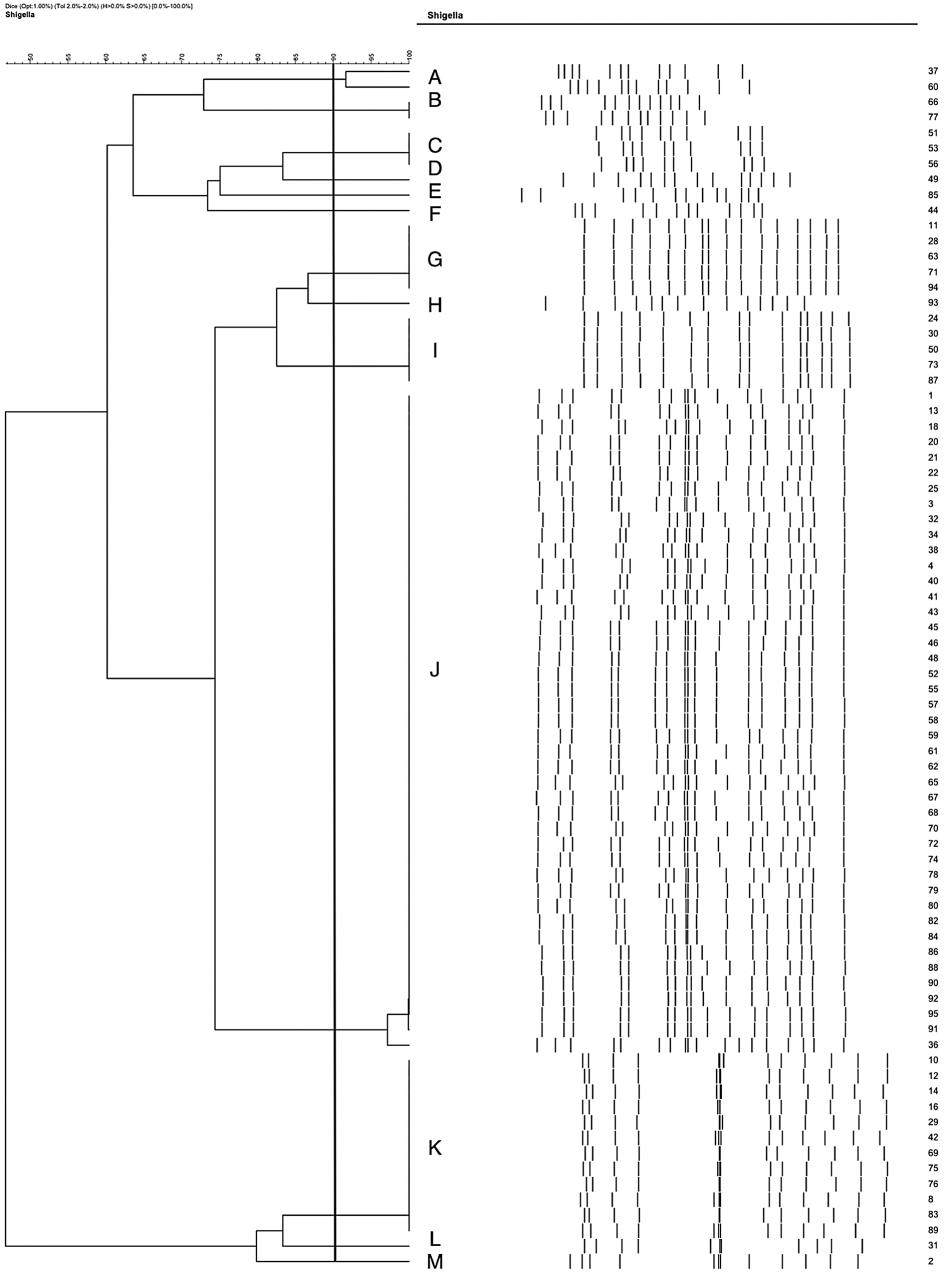
Dendrogram representing the PFGE profiles of 78 clinical Shigella isolates. The isolates were separated by 13 clusters. Isolates with 90% similarity (hypothetical red line) are placed inside a cluster. The effect of each antibiotic is mentioned in front of each isolate. C: chloramphenicol, AN: amikacin, IMP: imipenem, AZM: azithromycin, AM: ampicillin, TE: tetracycline, NA: nalidixic acid, FOX: cefoxitin, AMX: amoxicillin, FEP: cefepime, K: kanamycin, GM: gentamicin, NOR: norfloxacin, Sensitive, Intermediate, Resistance

All 80 *Shigella* isolates were genotyped by the PFGE technique (Fig. 1). Based on the number of bands observed on the dendrogram, the isolates were grouped in thirteen clusters (A-M). Also, the findings of PFGE analysis showed a genotypic similarity of 45% among the thirteen clusters (Fig. 2). Regarding the distribution of 43 isolates (53.7%) among these clusters, the majority of them were categorized under cluster J (*n* = 43). Among the isolates, 6 different clusters (M, L, H, F, E, D) were single clones, which considering their isolation in different years and also from different age groups, can indicate the clonal spread of this bacterium. Cluster A included samples from both studied areas in Tehran, which were placed in one cluster with 90% similarity. Cluster B and C included only samples from Milad Hospital in Tehran. The single clones of clusters D, E, and F are 83, 74, and 73% similar to cluster B, respectively. Cluster G included samples from all three studied areas from Tehran and Qazvin. The single clone of the H cluster has 85% overlap with the G cluster, which can indicate the derivation of the H single clone from the G cluster and its stability. Cluster I also includes samples from all three studied areas of Tehran and Qazvin cities. The band patterns of the three clusters G, H and I are close, and cluster J (dominant pattern) is 82% similar to clusters G, H, and I. One sample from cluster J had a lower band than samples from the same group. The last two cases can indicate significant changes and developments in the dominant pattern in twenty years. Cluster K includes samples of all three areas from Tehran and Qazvin cities. The single clones of clusters L and M are samples from Milad Hospital and are 83-85% similar to cluster K. The similarity between the PFGE patterns of clinical isolates indicated the role of environmental transmission in spreading the infection.

### Finding of antimicrobial susceptibility test

All *Shigella* isolates of this research were assessed separately for susceptibility to common clinically relevant antibiotics (Table 2). Out of 80 isolates, 70% showed resistance to amoxicillin (56/80), 67% were resistant to tetracycline (54/80), and 60% were resistant to ampicillin (48/80). Resistance to nalidixic acid and chloramphenicol were obtained at 25% (20/80) and 17.5% (14/80), respectively. 15% of isolates showed resistance to imipenem, cefepime, and azithromycin (12/80). Concerning other tested antibiotics 8.75% were resistant to amikacin (7/80), 2.5% were resistant to kanamycin and gentamicin (2/80), and 1.25% showed resistance to norfloxacin and cefoxitin (1/80). In addition, among eighty isolates, 27.5% (22/80) were determined as MDR. The most common MDR pattern was simultaneously resistance to chloramphenicol, ampicillin, tetracycline, and amoxicillin (Table 3).

**Table 2:** Drug resistance rate of *Shigella* isolates

Antibiotic	*Shigella* isolates Total (*n* = 80)	No. of isolates	Valid Percent	Cumulative Percent
**NOR**	1.25%	1	1.25	100
**GM**	2.5%	2	2.5	100
**K**	2.5%	2	2.5	100
**FEP**	15%	12	15	100
**AMX**	70%	56	70	100
**FOX**	1.25%	1	1.25	100
**NA**	25%	20	25	100
**TE**	67%	54	67	100
**AM**	60%	48	60	100
**AZM**	15%	12	15	100
**IPM**	15%	12	15	100
**AN**	8.75%	7	8.75	100
**C**	17.5%	14	17.5	100

NOR: norfloxacin, GM: gentamicin, K: kanamycin, FEP: cefepime, AMX: amoxicillin, FOX: cefoxitin, NA: nalidixic acid, TE: tetracycline, AM: ampicillin, AZM: azithromycin, IMP: imipenem, AN: amikacin, C: chloramphenicol

**Table 3:** Multidrug resistance profile of the *Shigella* isolates (*n* = 80)

Pattern no.	Antibiotic resistance patterns	No. of isolates	Pattern of PFGE	Province	Overall MDR isolates
1	C, AN, IPM, AZM, AM, TE, NA, AMX, FEP	1	G	Tehran	20/80 (25%)
2	AM, TE, NA, FOX, AMX, FEP, K, GM	1	J	Tehran	
4	C, IPM, TE, AM, NA, AMX, FEP	1	J	Qazvin	
5	C, AN, IPM, AM, TE, NA, AMX	1	L	Tehran	
7	AN, IPM, AZM, AM, TE, AMX	3	A, I,J	Tehran	
8	AZM, TE, AM, NA, AMX	1	J	Qazvin/Tehran	
9	AZM, TE, AM, AMX, FEP	1	J	Qazvin	
10	C, AM, TE, NA, AMX	1	J	Qazvin	
11	TE, NA, AMP, FEP	1	K	Tehran	
12	C, AM, TE, AMX	4	B, G,G, J	Tehran	
13	C, AM, TE, NA	1	K	Qazvin	
14	AM, TE, NA, AMX	1	J	Tehran	
15	AM, TE, FEP, K	2	J, M	Tehran	
16	AZM, NA, AMX	1	D	Tehran	

C: chloramphenicol, AN: amikacin, IMP: imipenem, AZM: azithromycin, AM: ampicillin, TE: tetracycline, NA: nalidixic acid, FOX: cefoxitin, AMX: amoxicillin, FEP: cefepime, K: kanamycin, GM: gentamicin, NOR: norfloxacin

## Discussion

The transfer of bacteria from various sources can result in clusters of colonization or infection in humans. Identification of clonal relationships between isolates is very important to determine the source and different routes of infection transmission. One of the key methods to understand these relationships is bacterial typing, which includes categorizing and distinguishing bacterial isolates based on their genetic traits. Microbial typing is a method utilized to recognize and differentiate microorganisms at the species or subspecies level, and divided into phenotypic techniques and genotypic techniques [19].

Several typing methods have been developed to assess the clonal relatedness of bacterial species; however, PFGE has been selected as a method of choice for typing *Shigella* spp [20]. PFGE is one of the genotyping techniques that entails the extraction of large DNA molecules post-restriction enzyme digestion, which offers improved reliability, reproducibility, and discriminatory power in comprehending microbial diversity and managing infectious diseases [12, 14, 21].

In this research project, a total of 80 samples of *Shigella* were collected from Tehran and Qazvin from 2002 to 2022 and confirmed with molecular identification of *ipaH*. Isolates were examined using the PFGE technique to uncover their genetic connections and pinpoint potential clonal clusters. Isolates with PFGE pattern similarities of 90% or higher were grouped into the same cluster to identify and categorize closely related isolates. Moreover, their sensitivity to 13 various antibiotics was examined to determine their resistance patterns and the occurrence of multidrug-resistant. By merging these approaches, the objective of the study is to improve understanding of the genetic diversity, dissemination of clones, and patterns of antibiotic resistance among *Shigella* samples in these areas during the designated timeframe.

The results of PFGE analysis showed a single major cluster (cluster J) with 43 members constituting 55.12% of total isolates. Most of the isolates in this cluster were isolated from Tehran province, which suggests the possible clonal dissemination of this particular clone throughout this region. The comparison of *Shigella* PFGE patterns from this study with those from Korea and India has shown high pulsotype similarity and identical antimicrobial resistance patterns, which suggests two possible explanations: 1-dissemination or importation of the identical clones in Asian countries, probably through human travel or migration; and 2-appearance of similar genotypes in different countries through genomic rearrangement events [14, 22]. These findings highlight the importance of considering local and global factors in the spreading antibiotic-resistant *Shigella* strains. International travel, migration, and the movement of people across borders can contribute to disseminating resistant strains. Additionally, the genetic plasticity of bacteria, including *Shigella*, allows them to adapt to changing environments and develop similar genetic traits independently in different geographical locations.

The results of the current research were compared with those of similar research from pulse field gel electrophoresis of *Shigella* bacteria in Iran and Palestine. The pattern of bands of *Shigella* bacteria in the current study was identical to the pattern of *Shigella* bacteria in the mentioned research up to more than 90% in Iran and 70% in Palestine and this criterion was compared. According to the review of similar research conducted in 2012 and 2019 in Iran and Palestine, we realized a dominant pattern similar to the dominant pattern of the present study, and this indicates the claim that a dominant and stable pattern has caused shigellosis over 20 years [14, 23]. Therefore, the stability of this model can have different reasons, which we will explain. The low infectious dose of *Shigella* bacteria (10–100 organisms) makes it easy for the bacteria to spread through small-scale transmission and personal contact [4]. Also, lack of adequate hygiene practices, especially among children, can exacerbate Shigellosis outbreaks in schools and other settings, making it difficult to control [24, 25]. The stability and spread of the dominant clone over 20 years could be due to successive transmission cycles, potentially facilitated by parents, hospital, and laboratory personnel through contact with contaminated hands. The spread of the dominant clone across geographical regions could be attributed to infected travellers or migration, indicating the importance of personal contact in disseminating the bacteria.

This study aims to analyze the antibiotic susceptibility profiles of 80 *Shigella* isolates. The outcomes confirmed high resistance rates to amoxicillin, tetracycline, and ampicillin (70%, 67%, and 60% respectively). Also, moderate resistance rates were determined for nalidixic acid and chloramphenicol (25% and 17.5%, respectively). In contrast, there were low resistance rates to imipenem, cefepime, azithromycin, amikacin, kanamycin, gentamicin, norfloxacin, and cefoxitin (ranging from 1.25 to 15%). Furthermore, around 27.5% of the isolates were classified as MDR, which means they had been resistant to three or more classes of antibiotics. Resistance to chloramphenicol, ampicillin, tetracycline, and amoxicillin were the maximum commonplace MDR samples observed in studied isolates. A study conducted by Majalan et al. in 2018 [26] reported a high prevalence of resistance to ampicillin, and chloramphenicol, among *Shigella* isolates from Tehran. The results of their study demonstrated a high level of resistance to both antibiotics, which is consistent with the findings of the present study. Also, a study by Mashouf et al. in 2006 [27] investigated the antibiotic resistance patterns of *Shigella* isolates from Iran, Tehran and found a high prevalence of resistance to ampicillin (89%) and tetracycline (83%), which is in line with the results of the present study. Also, the study revealed a high prevalence of resistance to chloramphenicol (90%) and nalidixic acid (51%) among *Shigella* isolates. In contrast, the present study observed moderate resistance to these two antibiotics. Similarly, a research paper by Moradi et al. in 2021 [28] reported no resistance against imipenem, meropenem, cefoxitin, norfloxacin, levofloxacin, azithromycin, and amoxicillin among 2742 *shigella* isolates. The isolates of this study also showed a low prevalence of resistance to norfloxacin and cefoxitin. These differences in resistance levels between studies may be attributable to factors such as variations in the study population, sample size, study period, and specific *Shigella* strains analyzed.

### Limitations

This study has several limitations that should be acknowledged. Firstly, the sample size may not accurately represent the entire *Shigella* population in Iran or even within the two studied provinces, which could affect the generalizability of the findings. Additionally, there was a lack of access to detailed clinical information, such as patient outcomes, treatment history, and risk factors, which are essential for a comprehensive interpretation of the observed antibiotic resistance patterns. The temporal scope of data collection, spanning two decades, may also limit the ability to capture changes in antibiotic resistance patterns over time. Furthermore, the findings may not be applicable beyond Tehran and Qazvin due to regional differences in epidemiology and environmental factors. Despite these limitations, this study provides valuable insights into the prevalence of antibiotic-resistant *Shigella* and underscores the need for ongoing surveillance and antibiotic stewardship to combat the spread of these resistant strains.

## Conclusion

The surge in antibiotic-resistant *Shigella* strains presents a concerning trend, posing a global challenge in treating shigellosis, including in Iran. This escalation in multi-drug resistance complicates the effective management of *Shigella* infections, emphasizing the pressing need for comprehensive measures against antibiotic resistance. Although specific data linking antimicrobial resistance and PFGE genotyping patterns in clinically derived *Shigella* strains is limited, methodologies such as PFGE offer valuable insights into the genetic diversity and relationships among *Shigella* strains. This information indirectly contributes to understanding patterns of antimicrobial resistance. However, additional research is imperative to delve into the specific correlation between resistance genes and PFGE genotyping patterns in *Shigella* strains.

## Data Availability

The datasets used and/or analysed during the current study are available from the corresponding author on reasonable request.
